# Feeding live yeast (*Saccharomyces cerevisiae*) improved performance of mid-lactation dairy cows by altering ruminal bacterial communities and functions of serum antioxidation and immune responses

**DOI:** 10.1186/s12917-024-04073-0

**Published:** 2024-06-07

**Authors:** Qian Zhang, Lifeng Ma, Xiaoqing Zhang, Hao Jia, Tana, Yu Guo, Jize Zhang, Jianlong Wang

**Affiliations:** 1 Key Laboratory for Mode Innovation in Forage Production Efficiency, Ministry of Agriculture and Rural Affairs, Institute of Grassland Research, Chinese Academy of Agricultural Sciences, Hohhot, 010010 Inner Mongolia China; 2 Inner Mongolia of Animal Disease Prevention and Control Institution, Hohhot, 010020 Inner Mongolia China; 3 National Center of Technology Innovation for Dairy, Hohhot, 010000 Inner Mongolia China

**Keywords:** *Saccharomyces cerevisiae*, Lactation performance, Ruminal microbiota-host interaction, Animal health

## Abstract

**Background:**

The utilization of live yeast (*Saccharomyces cerevisiae*, YE) in dairy cows is gaining traction in dairy production as a potential strategy to improve feed efficiency and milk yield. However, the effects of YE on dairy cow performance remain inconsistent across studies, leaving the underlying mechanisms unclear. Hence, the primary aim of this study was to investigate the impact of YE supplementation on lactation performance, ruminal microbiota composition and fermentation patterns, as well as serum antioxidant capacity and immune functions in dairy cows.

**Results:**

Supplementation with YE (20 g/d/head) resulted in enhancements in dairy cow’s dry matter intake (DMI) (*P* = 0.016), as well as increased yields of milk (*P* = 0.002) and its components, including solids (*P* = 0.003), fat (*P* = 0.014), protein (*P* = 0.002), and lactose (*P* = 0.001) yields. The addition of YE led to significant increases in the concentrations of ammonia nitrogen (NH_3_-N) (*P* = 0.023), acetate (*P* = 0.005), propionate (*P* = 0.025), valerate (*P* = 0.003), and total volatile fatty acids (VFAs) (*P* < 0.001) in rumen fermentation parameters. The analysis of 16s rRNA gene sequencing data revealed that the administration of YE resulted in a rise in the relative abundances of three primary genera including *Ruminococcus*_2 (*P* = 0.010), *Rikenellaceae*_RC9_gut_group (*P* = 0.009), and *Ruminococcaceae*_NK4A214_group (*P* = 0.054) at the genus level. Furthermore, this increase was accompanied with an enriched pathway related to amino acid metabolism. Additionally, enhanced serum antioxidative (*P* < 0.05) and immune functionalities (*P* < 0.05) were also observed in the YE group.

**Conclusions:**

In addition to improving milk performance, YE supplementation also induced changes in ruminal bacterial community composition and fermentation, while enhancing serum antioxidative and immunological responses during the mid-lactation stage. These findings suggest that YE may exert beneficial effects on both rumen and blood metabolism in mid-lactation dairy cows.

**Supplementary Information:**

The online version contains supplementary material available at 10.1186/s12917-024-04073-0.

## Background

High-yielding dairy cows during early and mid-lactation encounter challenges in utilizing diets rich in readily fermentable carbohydrates due to physiological limitations [1]. This can lead to a combination of inadequate rumen buffering from fine feed particles and reduced rumination time, ultimately disrupting microbial balance [2]. Consequences include decreased volatile fatty acids (VFAs) production, elevated lactic acid concentrations, and potentially subacute ruminal acidosis (SARA) [2]. Therefore, enhancing dairy cow well-being and productivity remains a key objective in modern dairy production research. The rumen, a complex ecosystem of microbes, plays a crucial role in the digestion and utilization of nutrient, significantly impacting overall cow performance [3]. The composition and function of the rumen bacterial community directly influence rumen fermentation, nutrient availability for the cow, and ultimately, milk yield [4]. Therefore, researchers are increasingly focused on investigating strategies to modulate the rumen microbiota.

Research on live yeast (*Saccharomyces cerevisiae*, YE) supplementation in dairy cow dates back to the 1950s and continues to be actively investigated [5]. YE is a rich source of nutrients, including vitamins, amino acids, peptides, minerals, organic acids, antioxidants, oligosaccharides, and β-glucans, which have been shown to promote the proliferation of rumen bacteria, protozoa, and fungi [6]. Growing evidence supports the effectiveness of YE supplementation in various aspects, including stimulating cellulolytic bacteria, enhancing the growth of lactate-utilizing bacteria, and mitigating post-feeding rumen pH decline [7]. Multiple studies have reported increased milk yield associated with YE or related products [8–10]. However, the observed responses vary significantly due to factors like yeast strain, dose, and mode of action, as well as animal factors such as lactation stage, production level, die composition (energy level), and parity [11]. The presence of these characteristics poses challenges in comparing outcomes and assessing YE efficacy on individual farms. Moreover, prior research has primarily focused on the effects of YE (*S. cerevisiae*) on rumen fermentation parameters and nutrient metabolism [12–15]. A few studies have investigated its specific impacts on the composition and functions of the bacterial population within the rumen [16, 17]. However, the findings from these studies regarding the interaction between ruminal microbiota and dairy cows have shown inconsistencies, leading to variations in microbiota composition and functions [18–20]. Therefore, we hypothesized that incorporating YE (*S. cerevisiae*) into diet of mid-lactation high-producing dairy cows would have positive effects on milk performance through its modulation of rumen microbial composition and function. Thus, the object of this study was to investigate the effect of YE supplementation on lactation performance, the rumen bacterial community and its predicted functions, as well as serum antioxidation and immune properties in mid-lactation dairy cows.

## Results

### Lactation performance

As displayed in Table 1, an evaluation of lactation performance revealed significant treatment effects. Dairy cows supplemented with YE had reduced dry matter intake (DMI) compared to the control (CON) group (*P* = 0.016), while milk yield significantly increased in the YE group (*P* = 0.002). Milk protein and lactose percentages were independently affected by treatment (*P* = 0.019) and time (*P* < 0.001). Importantly, YE treatment significantly improved the yield of milk fat, protein, lactose, and solids, as well as production efficiency measures such as 3.5% fat corrected milk (FCM) and energy corrected milk (ECM) (*P* = 0.014, *P* = 0.002, *P* = 0.001, *P* = 0.003, *P* = 0.001, and *P* < 0.001). It’s important to note that time did not exert a significant effect on these lactation performance variables, nor were there significant interactions between treatment and time observed for any measured parameters.

**Table 1:** DMI, milk yield, and composition of dairy cows fed the basal diet (CON) or addition of 20 g/d live yeast (YE).

Item	Treatment		SEM	*P*-value		
	CON	YE		Trt	Time	Trt×Time
DMI, kg/d	18.19^b^	19.03^a^	0.311	0.016	< 0.001	0.848
Milk yield, kg/d	32.79^b^	34.81^a^	0.560	0.002	0.524	0.484
Milk fat, %	3.84	4.07	0.110	0.051	0.337	0.169
Milk protein, %	3.04^b^	3.22^a^	0.040	0.019	0.036	0.549
Milk lactose, %	4.79	4.86	0.050	0.060	< 0.001	0.187
Milk solids, %	11.30	11.70	0.310	0.065	0.174	0.854
Milk fat yield, kg/d	1.26^b^	1.42^a^	0.074	0.014	0.345	0.238
Milk protein yield, kg/d	0.99^b^	1.13^a^	0.033	0.002	0.559	0.244
Milk lactose yield, kg/d	1.57^b^	1.72^a^	0.030	0.001	0.435	0.253
Milk solids yield, kg/d	3.71^b^	4.08^a^	0.136	0.003	0.160	0.562
3.5%FCM, kg/d	20.99^b^	23.27^a^	0.590	0.001	0.358	0.644
ECM, kg/d	34.67^b^	38.36^a^	0.890	< 0.001	0.528	0.272

DMI, dry matter intake; CON, control; YE, live yeast; Trt, treatment

3.5% FCM, fat corrected milk = (0.432 + 0.165 × kg milk fat) × kg milk yield

ECM, energy corrected milk = (0.327 × kg milk yield) + (12.95 × kg milk fat) + (7.65 × kg milk protein)

Data are presented as means and SEM, CON (*n* = 10), YE (*n* = 10). ^a,b^ Mean values in the same row with different superscript letters indicate a significant difference (*P* < 0.05). Trt = Treatment.

### Ruminal fermentation profiles

Rumen fermentation parameters (Table 2) showed no significant differences between the two groups for pH (*P* = 0.236), concentrations of butyrate (*P* = 0.062), isobutyrate (*P* = 0.069), isovalerate (*P* = 0.117), or the ratio of acetate to propionate (*P* = 0.6610). However, the YE group exhibited significantly higher concentrations of ammonia nitrogen (NH_3_-N), acetate, propionate, valerate, and total VFA (TVFA) compared to the CON group (*P* = 0.023, *P* = 0.005, *P* = 0.025, *P* = 0.003, and *P* < 0.001, respectively).

**Table 2:** Rumen fermentation parameters of dairy cows fed the basal diet (CON) and addition of 20 g/d live yeast (YE).

Items	CON	YE	SEM	*P*-value
pH	6.06	6.31	0.10	0.236
NH_3_-N, mg/dL	18.52^b^	22.77^a^	1.71	0.023
Acetate, mmol/L	51.60^b^	59.16^a^	2.34	0.005
Propionate, mmol/L	17.71^b^	19.86^a^	0.88	0.025
Butyrate, mmol/L	12.48	14.21	0.8	0.062
Isobutyrate, mmol/L	1.97	2.74	0.39	0.069
Valerate, mmol/L	0.97^b^	1.53^a^	1.16	0.003
Isovalerate, mmol/L	1.66	1.77	0.07	0.117
Total VFA, mmol/L	86.40^b^	99.26^a^	2.79	< 0.001
Acetate: propionate	2.93	3.01	0.17	0.661

CON, control; YE: live yeast; VFA, volatile fatty acid

Data are presented as means and SEM, CON (*n* = 10), YE (*n* = 10). ^a,b^ Mean values in the same row with different superscript letters indicate a significant difference (*P* < 0.05)

### Serum antioxidation and immune responses

As shown in Table 3, compared to the CON group, the YE group exhibited significantly increased activity of serum antioxidant enzymes, including catalase (CAT), glutathione peroxidase (GSH-Px), and superoxide dismutase (SOD) (*P* < 0.05). Additionally, the concentration of malondialdehyde (MDA), a marker of oxidative stress, was significantly lower in the YE group (*P* < 0.05). Regarding serum immune response, YE supplementation significantly increased the concentrations of immunoglobulin A (IgA), G (IgG), and M (IgM) compared to the CON group (*P* < 0.05). Nevertheless, the contents of soluble CD4 (sCD4) and soluble CD8 (sCD8) were significantly higher in the CON group compared to the YE group (*P* < 0.05).

**Table 3:** Serum antioxidation and immune response indices of dairy cows fed the basal diet (CON) and addition of 20 g/d live yeast (YE).

Items	CON	YE	SEM	*P*-value
Antioxidation indices				
CAT (U/mL)	65.17^b^	72.38^a^	2.43	0.002
GSH-Px (U/mL)	582.8^b^	727.1^a^	31.2	0.001
MDA (nmol/mL)	4.99^b^	4.02^a^	0.33	0.013
SOD (U/mL)	68.25^b^	75.99^a^	3.23	0.034
Immune response indices				
IgA (µg/mL)	21.35^b^	26.72^a^	0.58	< 0.001
IgM (µg/mL)	15.43^b^	22.84^a^	1.45	0.003
IgG (µg/mL)	225.6^b^	258.4^a^	3.87	0.009
sCD4 (U/mL)	35.15^a^	31.34^b^	0.83	< 0.001
sCD8 (U/mL)	19.19^a^	15.76^b^	1.08	0.008

CON, control; YE, live yeast, SEM, standard error of mean

CAT, catalase; GSH-Px, glutathione peroxidase; MDA, malondialdehyde; SOD, IgA, immunoglobulin A; IgM, immunoglobulin M; IgG, immunoglobulin G; sCD4, soluble CD4; sCD8, soluble CD8.

Data are presented as means and SEM, CON (*n* = 10), YE (*n* = 10). ^a,b^ Mean values in the same row with different superscript letters indicate a significant difference (*P* < 0.05)

### Ruminal bacterial communities

**Fig. 1:**
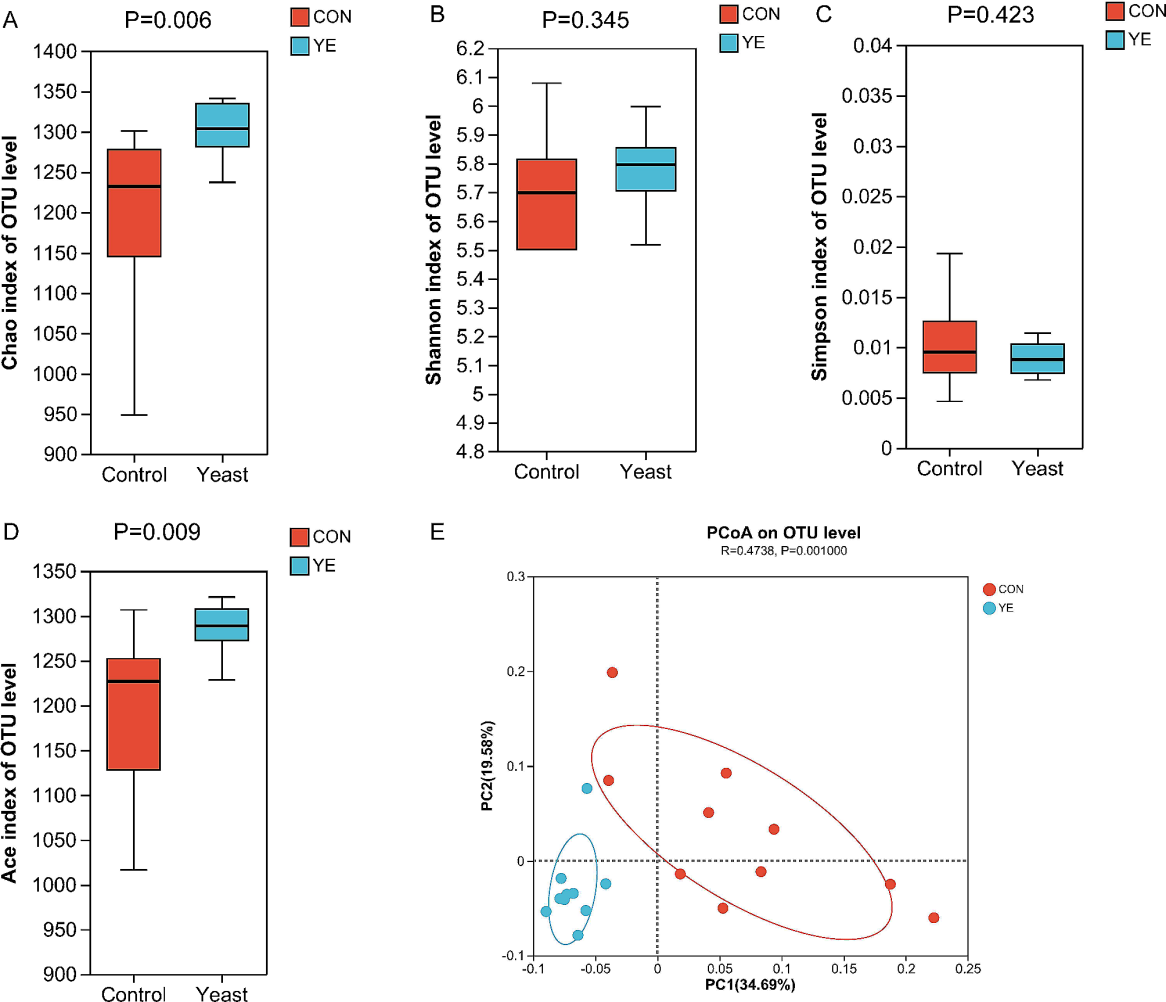
Effects of live yeast (YE) on ruminal microbiota of dairy cows. (**A**), (**B**), (**C**), and (**D**) The richness and diversity indices of rumen microbiota in dairy cows fed basal (CON) or live yeast (YE) diet. (**E**) Principal coordinate analysis (PCoA) of the overall rumen microbiota in dairy cows based on unweighted UniFrac distance

**Fig. 2:**
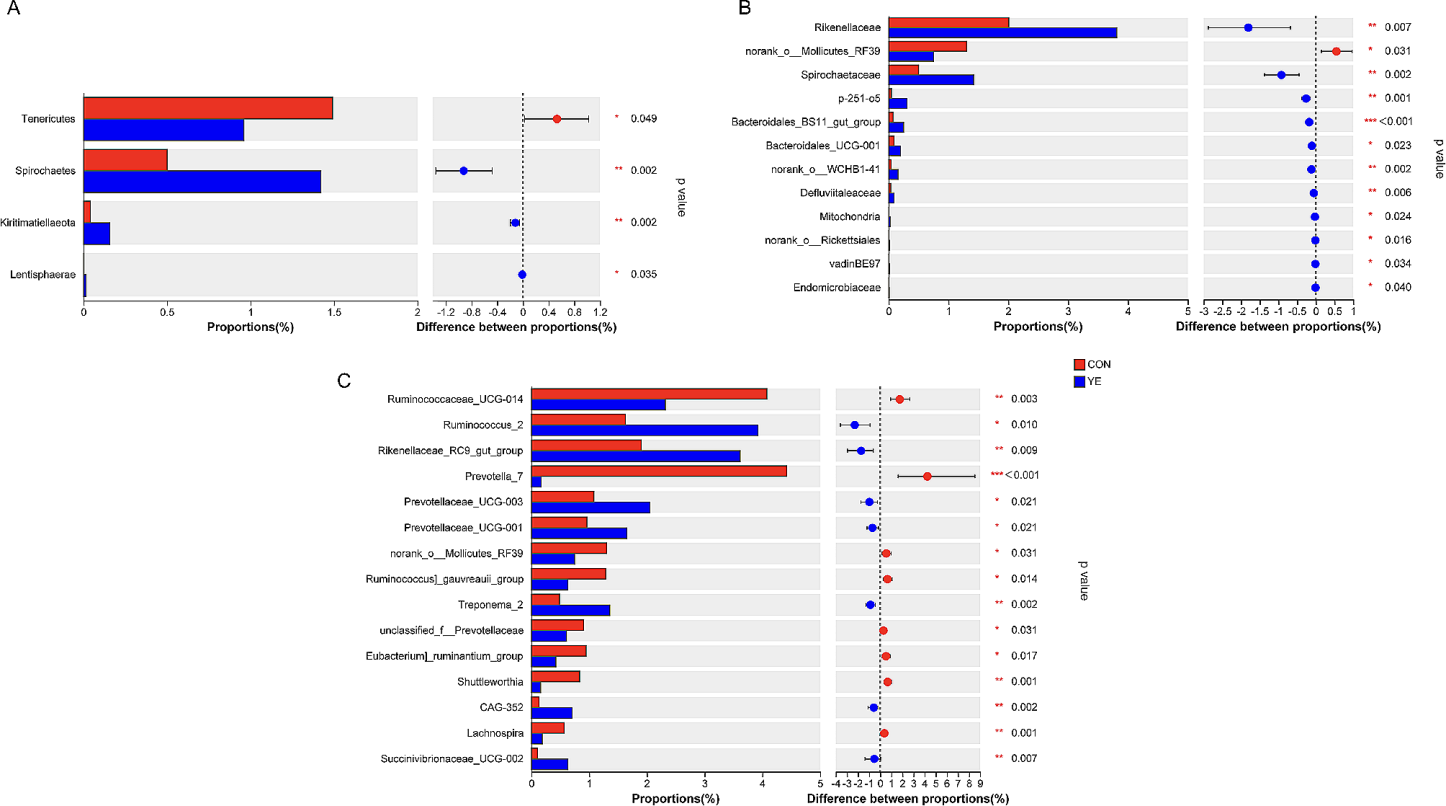
Effects of live yeast (YE) on the rumen bacterial composition at the phylum (**A**), family (**B**), and genus (**C) levels in dairy cows.** “*”, “**”, and “***” indicate the significance level at 0.05, 0.01 and 0.001, respectively

**Fig. 3:**
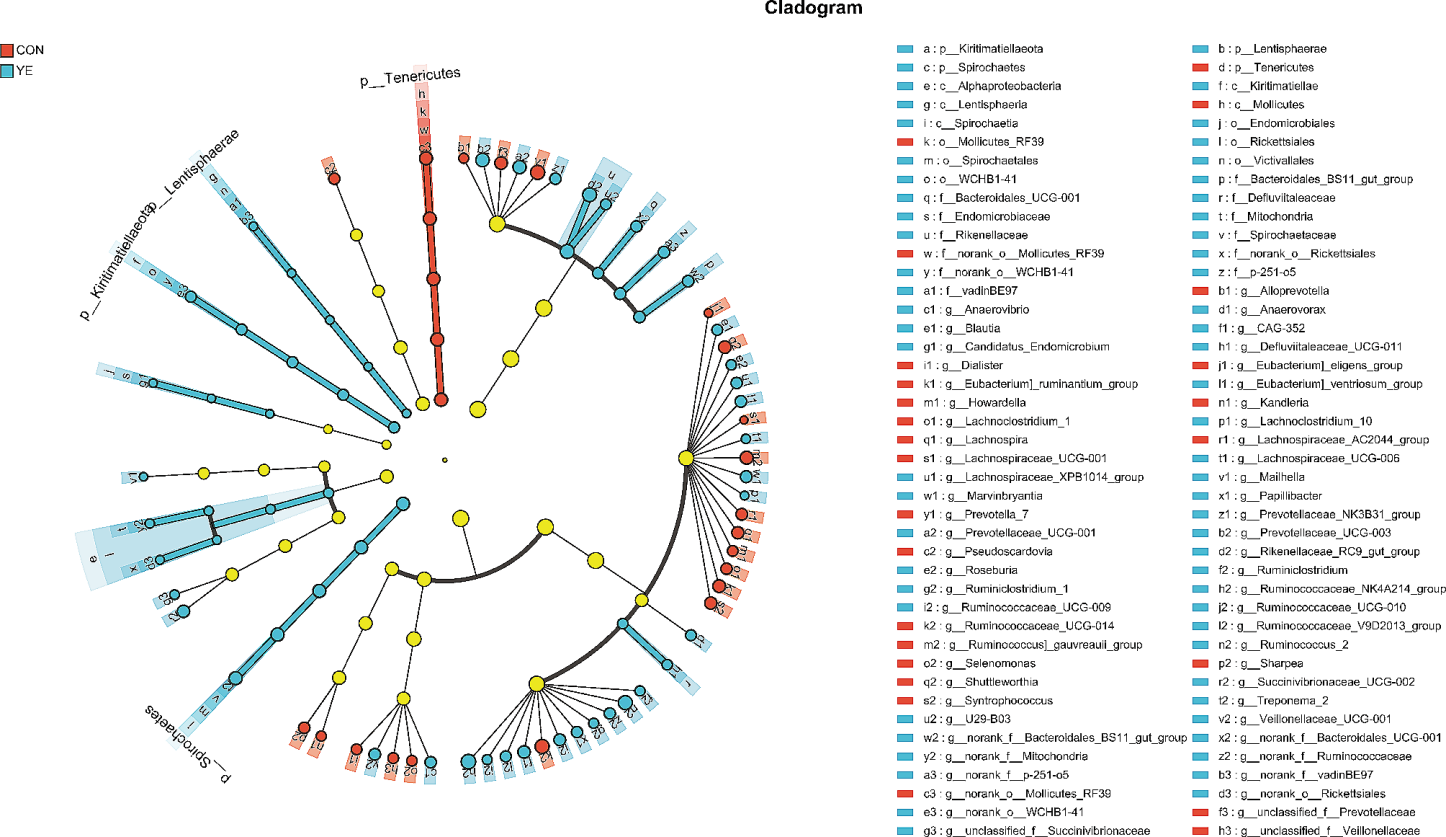
**LEFse (Liner discriminant analysis Effect Size) cladogram comparing microbiota communities between the YE and CON groups.** Differences are represented by colour, indicating the group where taxa are most abundant: red = taxa abundant in the basal (CON) group, blue = tax abundant in the live yeast (YE) group

Alpha diversity analyses revealed significant variations in multiple indices between the two groups. The Chao1 and Ace richness indices demonstrated a significant increase in the YE group in comparison to the CON group (Fig. 1a and d). Conversely, there were no significant variations seen in the Shannon and Simpson indices between the two groups (Fig. 1b and c). Beta diversity analysis indicated a significant difference (*P* = 0.001) in the rumen microbiota compositions between cows fed YE and those in the CON group (Fig. 1e). The taxonomic study provided annotations for a total of 17 bacterial phyla, specifically at the phylum level. The phyla *Firmicutes* and *Bacteroidetes* were found to be the most abundant, representing 45.98-46.95% and 44.95-46.12% of the total sequences, respectively (Supplementary file 1). The taxonomic groups *Patescibacteria*, *Actinobacteria*, *Proteobacteria*, *Tenericutes*, and *Spirochaetes* accounted for 1.54-2.57%, 1.11-2.27%, 1.12-1.48%, 0.96-1.49%, and 0.50-1.42% of the overall sequences, respectively. The CON group had higher abundance of *Tenericutes* in this study (*P* = 0.049). YE addition significantly increased the abundance of *Spirochaetes* (relative abundance > 0.5%, *P* = 0.002) (Fig. 2a). The dominating families at the family level were *Prevotellaceae* (33.76-36.9%), *Ruminococcaceae* (18.00-22.24%), and *Lachnospiraceae* (14.51-15.37%). Additional families observed in the study encompassed *Acidaminococcaceae* (4.17-5.26%), *Muribaculaceae* (2.65-3.90%), *Rikenellaceae* (2.01-3.81%), *Christensenellaceae* (2.13-2.72%), unidentified F082 (2.09-2.49%), *Veillonellaceae* (1.21-1.62%), and *Erysipelotrichaceae* (0.47-2.09%) (Supplementary file 1). The CON group had higher abundance of *norank_o__Mollicutes_RF39* in this study (*P* = 0.031). YE addition significantly increased the abundance of *Spirochaetaceae* (relative abundance > 0.5%, *P* = 0.002) and *Rikenellaceae* (*P* = 0.007) in dairy cows (Fig. 2b). At the taxonomic level of genus (as shown in Supplementary file 1), the dominant genera observed in dairy cows were *Prevotella*_1, *Ruminococcaceae*_NK4A214_group, *Lachnospiraceae*_NK3A20_group, *Succiniclasticum*, norank_f__*Muribaculaceae*, *Ruminococcaceae*_UCG-014, *Ruminococcus*_2, *Rikenellaceae*_RC9_gut_group, *Christensenellaceae*_R-7_group, norank_f__F082, *Eubacterium_coprostanoligenes*_group, *Prevotellaceae*_UCG-003, and *Acetitomaculum*. These genera were considered dominant as their relative abundance exceeded 1%. The CON cows had higher abundances of *Prevotella*_7 (*P* < 0.001) and *Ruminococcaceae*_UCG-014 (*P* = 0.003). YE addition significantly increased the abundances of *Ruminococcus*_2 (*P* = 0.010) and *Rikenellaceae*_RC9_gut_group (*P* = 0.009) (Fig. 2c) and trended toward increasing the abundance of *Ruminococcaceae*_NK4A214_group in dairy cows (*P* = 0.054). The cladogram presented in Fig. 3 demonstrated the predominant microbiome structure and highlighted the notable variations in taxa between the CON and YE groups, as indicated by liner discriminant analysis effect size (Lefse) analysis. The results acquired from the study revealed that the CON group exhibited a higher abundance of 25 clades, whereas the YE group showed a higher abundance of 61 clades. The CON group had *Prevotella*_7 as the sole differential biomarker with an LDA score beyond 4. The differential biomarkers in the YE group, as indicated by an LDA score greater than 4, were *Ruminococcus*_2 and *Ruminococcaceae*_NK4A214_group (Supplementary file 2).

### The correlation between bacterial populations, lactation performance, and ruminal fermentation profiles

**Fig. 4:**
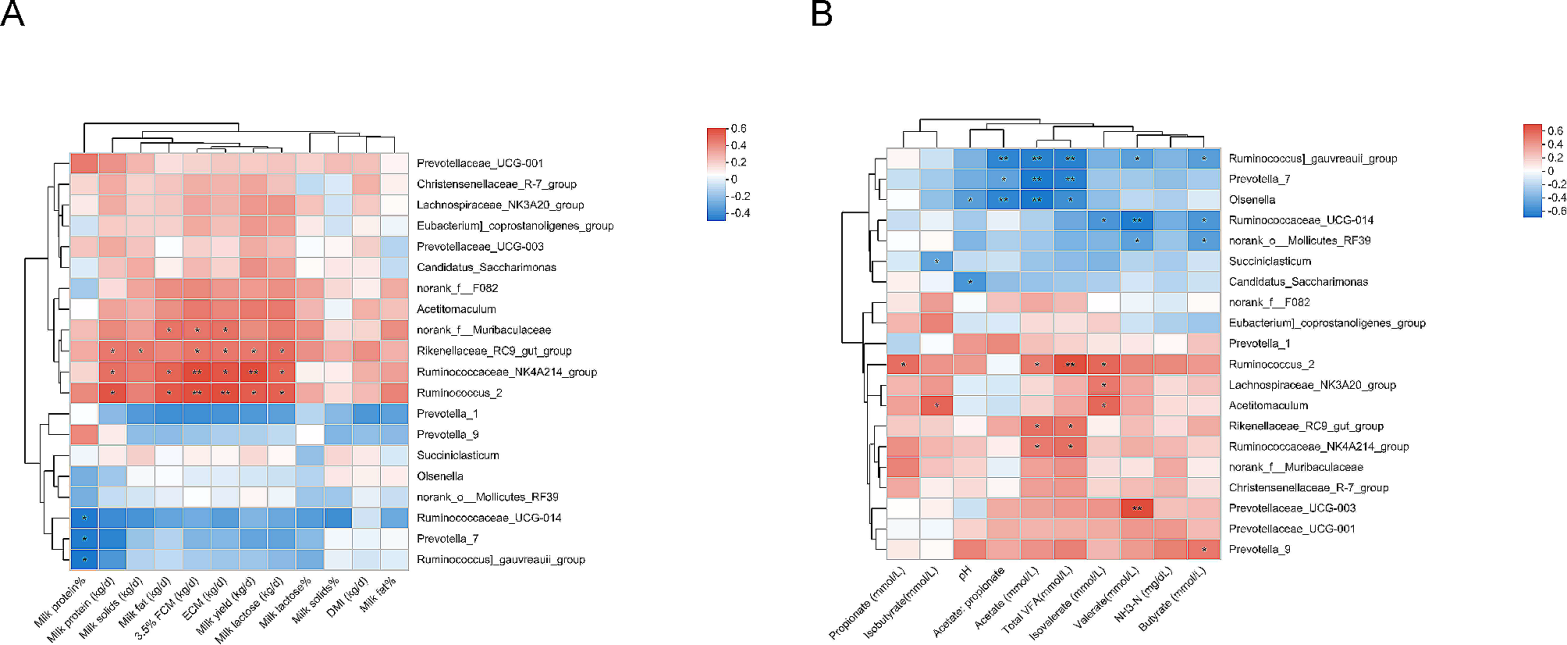
Correlation between the relative abundances of rumen bacteria, lactation performance (**A**), and fermentation parameters (**B**). “*”, “**”, and “***” indicate the significance level at 0.05, 0.01 and 0.001, respectively

Spearman’s rank correlation analysis revealed associations among lactation performance, ruminal fermentation characteristics, and the predominant ruminal bacterial populations in dairy cows (Fig. 4). As shown in Fig. 4a, there was a negative correlation observed between the relative abundances of *Ruminococcaceae*_UCG-014 (*r* = -0.470, *P* = 0.036), *Prevotella*_7 (*r* = -0.464, *P* = 0.040), and [*Ruminococcus*]*_gauvreauii*_group (*r* = -0.597, *P* = 0.033) with milk protein percentage. While milk protein (kg/d), milk yield (kg/d), and milk lactose (kg/d) exhibited positive correlations with the relative abundances of *Rikenellaceae*_RC9_gut_group (*r* = 0.451, *P* = 0.046; *r* = 0.449, *P* = 0.047; *r* = 0.476, *P* = 0.034), *Ruminococcaceae*_NK4A214_group (*r* = 0.473, *P* = 0.035; *r* = 0.579, *P* = 0.007; *r* = 0.501, *P* = 0.025), and *Ruminococcus*_2 (*r* = 0.554, *P* = 0.011; *r* = 0.525, *P* = 0.017; *r* = 0.519, *P* = 0.019). Similarly, there was a positive correlation between the yield of milk solids (kg/d) and the relative abundance of *Rikenellaceae*_RC9_gut_group (*r* = 0.446, *P* = 0.049). The study found a favorable correlation between milk fat (kg/d) and the relative abundances of norank_f__*Muribaculaceae* (*r* = 0.462, *P* = 0.040), *Ruminococcaceae*_NK4A214_group (*r* = 0.509, *P* = 0.022), and *Ruminococcus*_2 (*r* = 0.537, *P* = 0.015). The relative abundances of norank_f__*Muribaculaceae*, *Rikenellaceae*_RC9_gut_group, *Ruminococcaceae*_NK4A214_group, and *Ruminococcus*_2 were found to be positively correlated with the production efficiency of 3.5% FCM (kg/d) and ECM (kg/d) (*r* = 0.454, *P* = 0.044; *r* = 0.463, *P* = 0.040; *r* = 0.453, *P* = 0.045; *r* = 0.448, *P* = 0.048; *r* = 0.583, *P* = 0.007; *r* = 0.548, *P* = 0.012; *r* = 0.566, *P* = 0.009; *r* = 0.573, *P* = 0.008).

Analysis correlations between rumen fermentation parameters and bacterial populations revealed distinct associations (Fig. 4b). There was a positive correlation between the concentrations of TVFA and acetate, and the relative abundances of the genera *Ruminococcus*_2 (*r* = 0.610, *P* = 0.004; *r* = 0.450, *P* = 0.046), *Rikenellaceae*_RC9_gut_group (*r* = 0.474, *P* = 0.034; *r* = 0.485, *P* = 0.030), and *Ruminococcaceae*_NK4A214_group (*r* = 0.495, *P* = 0.026; *r* = 0.459, *P* = 0.042). Conversely, there was a negative correlation between the concentrations of TVFA and acetate, and the genera [*Ruminococcus*]*_gauvreauii*_group (*r* = -0.612, *P* = 0.004; *r* = -0.614, *P* = 0.004), *Prevotella*_7 (*r* = -0.618, *P* = 0.004; *r* = -0.652, *P* = 0.002), and *Olsenella* (*r* = -0.551, *P* = 0.012; *r* = -0.656, *P* = 0.012). Notably, there was a positive correlation observed between the concentration of propionate and the relative abundance of *Ruminococcus*_2 (*r* = 0.503, *P* = 0.024). Interestingly, there was a significant positive correlation between the concentration of valerate and the relative abundance of *Prevotellaceae*_UCG-003 (*r* = 0.627, *P* = 0.003). While there were significant negative correlations between the valerate concentration and the relative abundances of [*Ruminococcus*]_*gauvreauii*_group (*r* = -0.474, *P* = 0.035), *Ruminococcaceae*_UCG-014 (*r* =-0.636, *P* = 0.003), and norank_o__*Mollicutes*_RF39 (*r* = -0.469, *P* = 0.037).

### Function predictions of ruminal bacterial populations

**Fig. 5:**
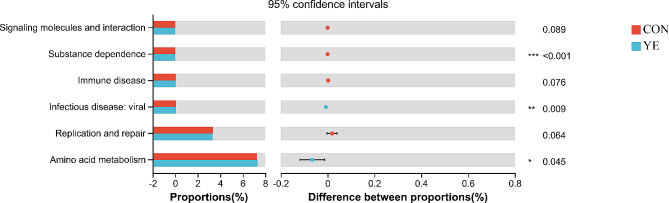
**Functional prediction of bacterial populations in ruminal samples of dairy cows fed the basal (CON) or live yeast (YE) diet.** Prediction of the differential function of rumen microbes between two groups of dairy cows in Kyoto Encyclopaedia of Genes and Genomes (KEGG) level 2 category based on PICRUSt 2. “*”, “**”, and “***” indicate the significance level at 0.05, 0.01 and 0.001, respectively

We performed functional predictions using phylogenetic investigation of communities by reconstruction of unobserved states 2 (PICRUSt2) to further understand the ruminal bacteria. All samples shared 46 predicted gene families. As shown in Fig. 5, the YE group exhibited a significantly higher relative abundances of gene families associated with the infectious disease (*P* = 0.009) and amino acid metabolism (*P* = 0.045) compared to the CON group. Conversely, the CON group had a significantly greater relative abundance of gene families linked to substance dependence (*P* < 0.001).

## Discussion

This study identified significant improvements in lactation performance within the YE group compared to the CON group. These included increased DMI, milk yield, and the yields of milk fat, protein, solids, and lactose. While previous research using YE supplementation in dairy cows reported similar individual variations in performance [21–23], the overall impact of YE remains variable across studies [11]. This inconsistency suggests a complex mechanism of action for YE. Multiple factors likely contribute to these differential reactions, including yeast strain, dosage, diet composition, lactation stage, and experimental design employed in various investigations [24]. Further research is crucial to elucidate the specific influence of these factors on YE’s effectiveness. Our findings demonstrate that cows supplemented with YE exhibited superior lactation performance, particularly in milk yield and its components (fat, protein, lactose, and solids). This difference in performance could be largely ascribed to the higher DMI and concentration of ruminal VFAs in the present study. Documented literatures support this connection, as YE supplementation has been shown to elevate DMI and apparent digestibility of nutrients in dairy animals, ultimately increasing digestible energy of the diet available for improved lactation performance [10, 25].

Previous studies highlighted the role of ruminal bacteria in enhancing the feed nutrient utilization efficiency [26], and their potential link to milk composition [27–29]. Our study, using 16sRNA gene sequencing, revealed differences in ruminal bacterial richness and diversity (measured by Chao1 and Ace indices) between the YE and CON groups. This aligns with proved evidence suggesting greater bacterial richness and diversity are associated with increased production of ruminal fermentation products, such as VFAs, amino acids, and glucose, all beneficial for milk production [30].

Milk solids components, including fat, protein, lactose, and minerals, is influenced by both feed intake and ruminal fermentation [31, 32]. The current study observed higher concentrations of most individual VFAs and TVFA in the YE group as compared to the CON group. These elevated VFA concentrations, likely due to increased DMI and fermentation rates, may contribute to the higher milk yield and its associated constituents (fat, protein, lactose, and solids) observed in the YE group. VFAs serve as primary energy sources for milk production, and the YE group exhibited a significant increase in acetate and propionate, which aligns with previous findings [30]. The current study also identified correlations between specific bacterial populations and the fluctuations in milk yield and its associated components. The YE group displayed a greater abundance of *Rikenellaceae*_RC9_gut_group, a prominent bacterial known to generate fermentation end product such as propionate, acetate and/or succinate [33]. This finding indicates a potential role for *Rikenellaceae*_RC9_gut_group in enhancing milk solids yield by possibly influencing the production of these key fermentation products, thus affecting milk major components. A previous investigation has indicated a positive correlation between the relative abundances of *Ruminococcaceae*_NK4A214_group and *Ruminococcus*_2 with milk solids production [30]. In this study, we observed a positive association between the relative abundances of these genera and milk solids yield. However, it is important to note that these correlations did not reach statistically significance (*P* = 0.058 for *Ruminococcaceae*_NK4A214_group and *P* = 0.065 for *Ruminococcus*_2). Notably, both genera, classified under the *Ruminococcaceae* family, were identified as distinctive biomarkers in the YE group by LEfse analysis. Furthermore, a positive correlation was observed between these genera and the production of milk fat and protein. The *Ruminococcaceae* family is recognized for its significant expertise in enzymatic degradation of complex plant substances, particularly cellulose, leading to the production of VFA that serve as energy resources for animals or other rumen-resident bacteria [34]. Acetate serves as a precursor for de novo lipogenesis, contributing to milk fat synthesis in mammary gland epithelial cells of bovine. Additionally, acetate acts as an energy source for microbial protein synthesis [35]. The present investigation revealed significant positive associations between acetate concentration and the relative abundances of *Rikenellaceae*_RC9_gut_group, *Ruminococcaceae*_NK4A214_group and *Ruminococcus*_2. Moreover, KEGG function prediction indicated enhanced amino acid metabolism, supporting by higher NH_3_-N concentration in the YE group, suggesting sufficient nitrogen resources for protein synthesis. *Treponema*_2, a prominent genus in the YE group with a relative abundance of 1.36%, is associated with tryptophan metabolism in healthy early lactation Holstein dairy cows [36]. These findings partially elucidated the underlying factors contributing to the higher yields of milk, solids, protein, and fat observed in the YE group compared to the CON group in our study, possibly due to a positive correlation with acetate concentration. Surprisingly, valerate, a key VFA found to be higher in high milk yield cows [37], exhibited a negative correlation with the abundances of [*Ruminococcus*]_*gauvreauii*_group and *Ruminococcaceae*_UCG-014 in the current study. Another investigation also showed that *Ruminococcaceae* and *Ruminococcus* had a negative correlation with valerate due to variations in fermentation process (specially protein fermentation) induced by cross feeding mechanisms [38]. Meanwhile, a previous study demonstrated a positive correlation of *Ruminococcaceae* _UCG-014 with valerate in marine fish [39]. The inconsistency between valerate concentration and these microbiota species or strains indicates the complexity of the interaction between microbiota and VFAs in the host, which requires further research to elucidate the relationships between these microbiotas and valerate. *Prevotella* is believed to have a significant involvement in the process of starch breakdown [40]. In the current investigation, *Prevotella*_7 exhibited differential biomarker characteristics exclusively in the CON group, with its relative abundance decreasing following YE supplementation, consistent with previous reports [41, 42]. *Prevotella*_7 was found to be more abundant in cows with lower efficiency and milk yield, as well as moderate SARA [42–44]. Nevertheless, other studies have reported that elevated or unchanged levels of *Prevotella*_7 following YE supplementation [45, 46]. Further investigation is needed to elucidate the ruminal bacterial community’s responds to YE supplementation.

High concentrate diets in dairy cow are linked to metabolic and systemic dysfunction [47]. Inadequate management practices in commercial dairy farm can further lead to milk production-related diseases such as mastitis and metritis [47, 48]. These factors collectively induce oxidative stress and compromised immune function in dairy cows. Nutritional interventions have been shown to reduce pro-oxidant loads and decrease the incidence of oxidative stress through antioxidant mechanisms. In the current investigation, the serum activities of CAT, GSH-Px, and SOD in the YE group exhibited a significant increase (*P* < 0.05) compared to the CON group. In contrast, MDA concentrate was significantly reduced, indicating the great potential of YE in mitigating oxidative stress in dairy cows. Numerous studies have reported that both YE and its culture products contribute to increased serum activities of antioxidant enzymes, along with enhanced antioxidant capacity involving radicals scavenging and metal chelating activities [49, 50]. The potential antioxidative effects of YE may be attributed to its antioxidative compounds, including vitamins A, E and C, polysaccharides, and sulfur-containing amino acids [51]. Moreover, YE supplementation appeared to affect the serum levels of immunoglobulins (IgA, IgG, and IgM), as well as sCD4, and sCD8 levels during the study period. Immunoglobulin levels experienced a significant increased, whereas sCD4, and sCD8 levels exhibited a notable decrease. YE and its associated products contain various immunomodulating chemicals that engage in direct and indirect interactions with pathogens and components of the immune system [52, 53]. Notably, polysaccharide β-glucan, a key component of YE, is categorized as a biological response modifier [54]. It stimulates innate immunity by enhancing the function of macrophages and neutrophils [55] and promotes a robust adaptive immune response by increasing antibody production [56]. sCD4 and sCD8 are considered as markers of T lymphocyte activation, which are maintained during the inflammatory process [57]. The induction of SARA can be triggered by a high concentrate diet, which facilitates the pathogenesis of ruminal acidosis by releasing lipopolysaccharides (LPS) into the rumen fluid [58]. Some proportion of LPS can translocate into body’s circulation system through both paracellular and transcellular pathways, leading to an increase in sCD4 and sCD8 levels [59]. The findings of this study indicate that YE supplementation has the potential to enhance cow organism immunity and mitigate inflammation-induced damage to the immune system.

## Conclusions

In summary, our results demonstrated that the supplementation of YE to the diet increased DMI and lactation performance, along with enhanced serum antioxidative and immune functionalities in mid-lactation dairy cows. The application of YE also led to modifications in the ruminal microbiota and fermentation processes, resulting in improved amino acid metabolism. These findings significantly contribute to enhancing our overall understanding of the impacts of YE on rumen and blood metabolism in mid-lactation dairy cows.

## Methods

### Animal, diets, and experimental design

The current investigation was carried out in accordance with the Regulations for the Administration of Affairs Concerning Experimental Animals of the State Council of the People’s Republic of China. The research protocol employed in this study was granted approval by the Committee on Experimental Animal Management of the Chinese Academy of Agricultural Sciences, (Beijing), with the reference No. 39/14.08.2019. All methods were carried out in accordance with relevant guidelines and regulations and were reported in accordance with ARRIVE guidelines for the reporting of animal experiments.

The present study was carried out at Chaoren Dairy Co., in Yuncheng, Shanxi Province. Twenty mid-lactation, multiparous Chinese Holstein cows (mean weight 651 ± 12.47 kg) with similar days in lactation (110 ± 8) and overall health, were randomly assigned to two groups (*n* = 10) using a computer-generated randomization table. The cows were housed in individual tie-stall barns equipped with overhead fan devices situated above the lying area to ensure free access to water throughout the experiment. The control group (CON) received a total mixed ration (TMR) without yeast as the basal diet, whereas the treatment group (YE) was fed the same TMR supplemented with 20 g/d/cow of YE (strain Y03-0, 2.0 × 10^10^ CFU/g, ANGEL YEAST Co., Ltd., Yichang, China). The constituents and chemical composition of TMR as basal diet are summarized in Table 4. Feed was provided to the cows twice daily, at 0900 h and 1500 h, and milking procedures were conducted three times a day (0930 h, 1230 h, and 1530 h). The YE supplements was top-dressed on the TMR after initial consumption, followed by the remaining basal diet. The trial lasted for 60 d, divided into three 20-d periods. The experiment began with a 10-d adaption period, followed by a 50-d treatment period with YE. Feed intake measurements were conducted during the final two days of each experimental period. Feed was quantified at each feeding event, and the remaining amount was assessed prior to the morning feeding on the second day. DMI was calculated by subtracting the amount of feed delivered from the amount of residues, both measured on an absolute dried basis. Feed efficiency was determined using 3.5% of FCM and ECM. The nutritional composition of the feed was assessed on a per-period basis using the methods outlined by AOAC (2007). The feed samples obtained from each feeding session within the last 2 d of each experimental period were combined, and a total of 200 g of mixed feed was prepared for subsequent analysis. Detailed descriptions of feed sample analyses are provided in a prior publication [45].

**Table 4:** Ingredient and chemical composition of the basal diet

Items	Value
Alfalfa hay	13.8
Oat hay	2.30
Corn silage	45.20
Corn grain	14.58
Soybean meal	3.18
Cottonseed meal	3.18
Dry distillers grains with solubles	2.04
Wheat bran	1.17
Ca(HCO_3_)_2_	0.24
Limestone	0.30
NaHCO_3_	0.36
Premix^1^	1.44
Cottonseed	5.1
Pelleted sugar beet pulp	3.6
Total	100.00
Chemical composition^2^	
Dry matter (% as fed)	
NE_L_, Mcal/kg of DM	1.59
Crude protein	18.1
Ether extract	4.6
Neutral detergent fiber	38.2
Acid detergent fiber	24.0
Ash	7.12
Calcium	0.8
Total phosphorus	0.4

^1^One kilogram of premix contained the following: vitamin A 19.4–28.7 KIU, vitamin D 4.7–7.1 KIU, vitamin E 0.14–1.19 KIU, Cu 485–730 mg, Mn 1285–1925 mg, Zn 2210–3310 mg

^2^The chemical composition were measured values; NE_L_ was calculated according to NRC (2001) equations

### Sample collection and analysis

Milk yield and composition were determined on the two consecutive days every period throughout the experimental trial. Milk yield was recorded using the MagStream meter (BouMatic, Madison, WI, USA). The milk composition was measured by a near-infrared absorption analyzer (MilkoScan FT2, Foss Electric, Denmark). A detailed description of milk sample collection and composition analyzes are provided in a previous study [45].

On the final day of the formal experiment, two hours post-feeding in the morning, rumen fluid (100 mL) was collected via oral stomach sampling tube for each cow [49]. Each cow’s rumen fluid sample was snap-frozen in liquid nitrogen, with approximately 5 mL being used. These samples were then stored at -80℃ for the purpose of conducting 16 S rRNA sequencing. The pH of the remaining collected rumen fluid samples was determined using a portable pH meter (Seven2GO S7, Mettler-Toledo, Columbus, OH, USA). Subsequently, the samples were filtered through four layers of cheesecloth. The filtrate was preserved at -20℃ in order to facilitate the examination of rumen fermentation characteristics. These parameters encompassed VFA profiles, which were analyzed using gas chromatography (Agilent 6850, Agilent Technologies Inc., Santa Clara, CA, USA), as well as NH_3_-N concentrations [7].

Blood samples (*n* = 10) were collected at 6 h post-feeding (1500 h) via venipuncture of the tail vein and into evacuated tubes. In this study, blood samples of 10 mL each were collected and placed in tubes. These tubes were then allowed to reach room temperature and were held in this condition for a duration of 30 min. Subsequently, the tubes were centrifuged at *3000 g* for 10 min at 4℃. The serum samples were carefully stored at 20℃, awaiting further analysis. The immune response and antioxidation parameters of the serum were assessed using a fully automatic biochemistry analyzer (Hitachi 7020, Tokyo, Japan) to determine the levels of IgA, IgM, and IgG. Additionally, the levels of sCD4, sCD8, CAT, GSH-Px, MDA, and SOD were measured using a commercially available diagnostic kit (Nanjing Jiancheng Bioengineering Institute, Nanjing, China) in accordance with the manufacturer’s instructions.

### Microbial DNA extraction, sequencing and data analysis

The extraction of microbial DNA from rumen content samples (n = 10) was performed using the HiPure Stool DNA Kits (Angen, Guangzhou, China). The purity of the extracted DNA was assessed using the NanoDrop2000 spectrophotometer (Thermo Scientific, Madison, WI, USA). To generate separate amplicon libraries, the 16S rRNA V3-V4 region was amplified by PCR with the primer pair 341F (5’-CCTACGGGNGGCWGCAG-3’) and 806R (5’-GGACTACHVGGGTATCTAAT-3’). The sequencing process was conducted at a commercial laboratory (Majorbio Biotechnology Co., Ltd., Shanghai, China) using Illumina HiSeq 2500 platform, following established protocols. The raw data underwent processing through the use of quality filters in QIIME. Subsequently, the raw reads underwent a trimming process wherein adapters and low-quality sequences, possessing a quality score above 20, were removed. The study employed UPARSE (version 9.2.64) to conduct operational taxonomic unit (OTU) cluster analysis, using a similarity threshold of 97% as described by Edgar (2013) [60]. The taxonomic categorization of the typical OUT sequences into organisms was conducted using the RDP classifier, employing a confidence threshold value of 0.8. This classification was based on the SILVA database [61]. Bacterial diversity was evaluated using alpha diversity indices (Chao1, ACE, Simpson, and Shannon), and statistical analysis employed the Kruskal-Wallis test with False Discovery Rate (FDR) correction. The analysis of beta diversity was conducted by employing unweighted UniFrac distance metrics and principal coordinates analysis (PCoA) in order to evaluate the differences in bacterial communities across the various samples. The present study utilized LEfSe analysis to identify microbiota biomarker features in each group, utilizing the Kruskal-Wallis test for screening purpose. Additionally, the threshold for the LDA score was set to its default value of 2.0. It was observed that there was a statistically significant difference in the relative abundance between the two groups, as shown by an LDA value greater than 4.

The functional alternations of the microbiota in different samples were predicted through the utilization of PICRUSt2 analysis. This analysis was conducted based on the level 2 pathways of the Kyoto Encyclopedia of Genes and Genomes (KEGG) database. For more information on PICRUSt2, please refer to the following link: https://github.com/picrust/picrust2.

### Statistical analysis

The lactation performance data, including DMI, milk yield, and components, were subjected to analysis using the MIXED model of SPSS 20.0 software (SPSS Inc., Chicago, IL, USA). The analysis was conducted for a completely randomized design, with adaption period as a covariate factor. The analysis used fixed effects for treatment, period (time of sample collection), the interaction between treatment and period, and covariate, with the cow serving as the experimental unit. The residual (co)variance matrix was considered to have a compound symmetry structure. The AR1 covariance structure was employed in accordance with the methodology outlined in a prior research study [7]. Before analysis, normality of variance in the data was checked, and it was determined that no data changes were necessary. The data pertaining to rumen fermentation parameters and serum chemistry were subjected to analysis using the Student’s *t* test. The nonparametric test (Kruskal-Wallis) was employed to assess the variations in the relative abundance of bacterial communities. The significance level was determined to be *P* < 0.05. The statistical analysis to determine the relationship between lactation performance, rumen VFA concentrations and bacteria abundance was conducted using Spearman’s correlation test in SPSS 20.0. A significant level of *P* < 0.05 and a correlation coefficient (*r*) with an absolute value greater than 0.8 were considered indicators of significant correlations.

### Electronic supplementary material

Below is the link to the electronic supplementary material.

Supplementary Material 1

Supplementary Material 2
Additional file 1, Figure S1 Comparison of ruminal bacteria in dairy cows fed basal (CON) or live yeast (YE) diet. (A) Relative abundances of bacterial communities at the phylum level. (B) Relative abundances of bacterial communities at the family level. (C) Relative abundances of bacterial communities at the genus level.

Supplementary Material 3
Additional file 2, Figure S2 LDA value distribution histogram. LDA value > 4, and the length of the bar chart represents the influence of different species.

## Data Availability

The datasets generated and/or analyzed during the current study are available in the Sequence Read Archive of the National Center for Biotechnology database repository with accession project number PRJNA1034486.
