# Supplementary figures and images for: Feeding live yeast (Saccharomyces cerevisiae) improved performance of mid-lactation dairy cows by altering ruminal bacterial communities and functions of serum antioxidation and immune responses
Source: BMC Vet Res. 2024 Jun 7;20:245. doi: 10.1186/s12917-024-04073-0 (PMC11157803; doi:10.1186/s12917-024-04073-0)

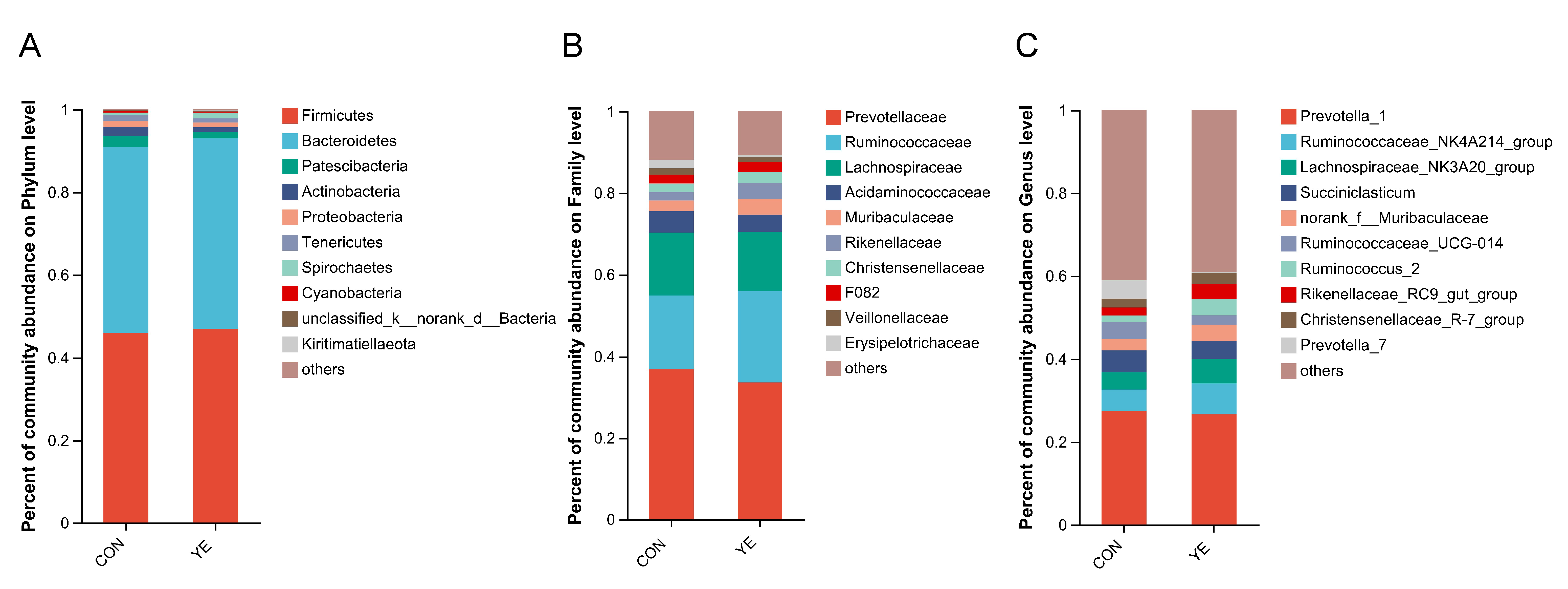

Supplement: Supplementary file 2 — Supplementary Material 2 Additional file 1, Figure S1 Comparison of ruminal bacteria in dairy cows fed basal (CON) or live yeast (YE) diet. (A) Relative abundances of bacterial communities at the phylum level. (B) Relative abundances of bacterial communities at the family level. (C) Relative abundances of bacterial communities at the genus level. [file 12917_2024_4073_MOESM2_ESM.tiff]

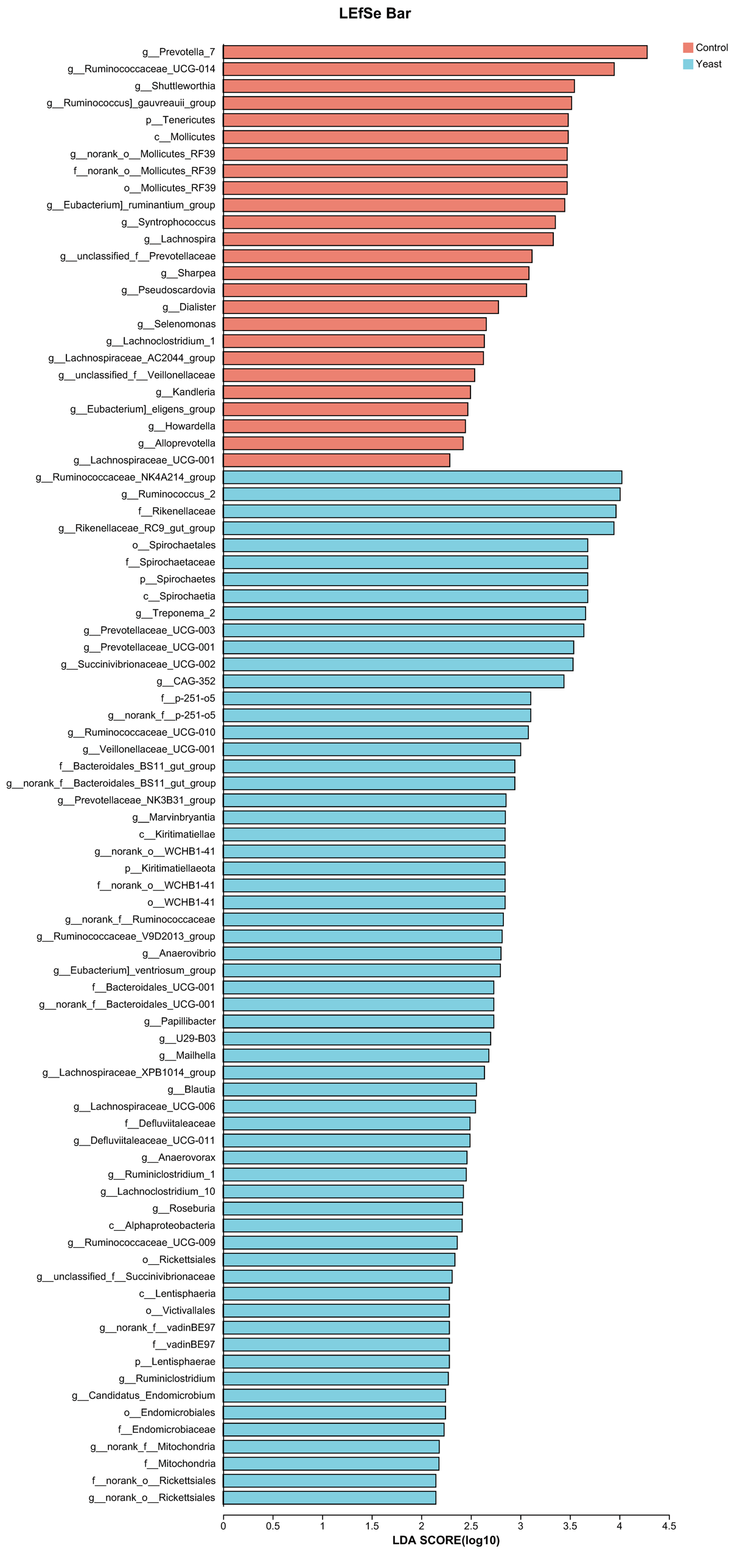

Supplement: Supplementary file 3 — Supplementary Material 3 Additional file 2, Figure S2 LDA value distribution histogram. LDA value > 4, and the length of the bar chart represents the influence of different species. [file 12917_2024_4073_MOESM3_ESM.tiff]
